# Enhanced Stim1 expression is associated with acquired chemo-resistance of cisplatin in osteosarcoma cells

**DOI:** 10.1007/s13577-017-0167-9

**Published:** 2017-03-22

**Authors:** Xilong Sun, Qiang Wei, Jie Cheng, Yanzhu Bian, Congna Tian, Yujing Hu, Huijie Li

**Affiliations:** 1 Department of Orthopaedics, Hebei General Hospital, Shijiazhuang, People’s Republic of China; 2 Department of Nuclear Medicine, Hebei General Hospital, Shijiazhuang, People’s Republic of China; 3 Department of Stomatology, Hebei General Hospital, Shijiazhuang, People’s Republic of China; 4 Department of Orthopedics, The Third Hospital of Hebei Medical University, No. 139, Ziqiang Road, Qiaoxi District, Shijiazhuang, 050051 Hebei People’s Republic of China

**Keywords:** Cisplatin resistance, Osteosarcoma, Endoplasmic reticulum, Apoptosis, Stim1

## Abstract

Osteosarcoma is the most common primary malignant bone tumor. Although cisplatin is the primary chemotherapy used in osteosarcoma treatment, the cisplatin resistance remains a big challenge for improving overall survival. The store-operated calcium (Ca^2+^) entry (SOCE) and its major mediator Stim1 have been shown to be implicated in a number of pathological processes typical for cancer. In this study, we showed that Stim1 expression was significantly increased in chemo-resistant osteosarcoma tissues compared with chemo-sensitivity tissues. Patients with Sitm1 expression exhibited poorer overall survival than Stim1-negative patients. Moreover, un-regulation of Stim1 expression and SOCE were also observed in cisplatin-resistant MG63/CDDP cells compared with their parental cells. Cisplatin treatment obviously reduced Stim1 expression and SOCE in cisplatin-sensitivity MG63 cells, but had no effects on MG63/CDDP cells. In addition, cisplatin resulted in a more pronounced increase of endoplasmic reticulum (ER) stress in MG63 cells than in their resistant variants, which was evidenced by the activation of molecular markers of ER stress, GRP78, CHOP and ATF4. Knockdown of Stim1 using siRNA remarkably enhanced cisplatin-induced apoptosis and ER stress in MG63/CDDP cells, thereby sensitizing cancer cells to cisplatin. On the other hand, overexpression of Stim1 markedly reversed apoptosis and ER stress following cisplatin treatment. Taken together, these results demonstrate that Stim1 as well as Ca^2+^ entry contributes cisplatin resistance via inhibition of ER stress-mediated apoptosis, and provide important clues to the mechanisms involved in cisplatin resistance for osteosarcoma treatment. Stim1 represents as a target of cisplatin and blockade of Stim1-mediated Ca^2+^ entry may be a useful strategy to improve the efficacy of cisplatin to treat osteosarcoma.

**Electronic supplementary material:**

The online version of this article (doi:10.1007/s13577-017-0167-9) contains supplementary material, which is available to authorized users.

## Introduction

Osteosarcoma is the most common primary malignant bone tumor in children and adolescents [1]. It accounts for about 2.4% of all malignancies in pediatric patients and 20% of primary bone cancer [2]. Patients with osteosarcoma will receive a three-drug chemotherapy regimen consisting of cisplatin, doxorubicin and methotrexate, followed by surgical resection of the primary tumor in which higher survival rates have been achieved [3, 4]. However, the development of chemo-resistance is a serious problem that largely limits the efficacy of chemotherapy. While many efforts have been made in the past [5, 6], little is known about the molecular mechanisms underlying osteosarcoma chemo-resistance for clinical therapy.

Multiple studies have emphasized the critical role of calcium (Ca^2+^) and Ca^2+^ ion channel in the regulation of cancer cell death and apoptosis [7–10]. Store-operated Ca^2+^ entry (SOCE) has been confirmed as the major Ca^2+^ influx pathway in non-excitable cells [11, 12]. Stim1, located in the membrane of endoplasmic reticulum (ER), is major component of store-operated Ca^2+^ channels and servers as a sensor of ER Ca^2+^ level [11, 13]. To note, previous studies have suggested a critical role of Stim1 in cancerogenesis. Knockdown of Stim1 or pharmacological inhibition of SOCE suppressed tumor metastasis in animal models of breast cancer [14]. Moreover, blockade of Stim1 inhibited hepatocarcinoma cell migration and sensitized prostate cancer cells to therapy [15]. However, the possible role of Stim1 in osteosarcoma is still inconclusive.

The ER is an essential organelle that is critical for the biological processes required for cell survival and death [16]. Upon multiple stimuli, unfolded and incompletely folded proteins are accumulated in ER, leading to unfolded protein response and ER stress [16, 17]. Importantly, ER plays a crucial role in Ca^2+^ homeostasis and Ca^2+^ signaling [18]. Moreover, previous studies have reported that ER stress can be induced by cisplatin and evidenced by the activation of molecular markers of ER stress, such as GRP78, CHOP and ATF4 [19, 20]. Considering the important role of Stim1 and SOCE in ER stress and apoptosis [18, 21], we proposed that Stim1 and SOCE might be involved in cisplatin resistance in cancer cells.

Therefore, in the present study, we explored the correlation between Stim1 and ER stress under cisplatin treatment in osteosarcoma cells, demonstrating that increased Stim1 as well as Ca^2+^ entry contributes to cisplatin resistance by inhibition of ER stress-mediated apoptosis. Our findings suggest that targeting Stim1 may be a potential strategy to improve the efficacy of chemotherapy for osteosarcoma.

## Methods and materials

### Patients and specimen preparation

The specimens of tumor tissues were collected from patients with osteosarcoma who underwent surgical resection between 2010 and 2013 in the Department of Orthopedics of The Third Hospital of Hebei Medical University. These patients were received standardized protocol consisting of neoadjuvant chemotherapy followed by appropriate surgical management. The specimens from 60 cases of osteosarcoma were divided into the following groups based on the follow-up visits from 2011: (1) subjects with a good response and a survival of more than 3 years (*n* = 28); (2) subjects with a poor response, who died within 3 years because of requiring chemo-resistance (*n* = 32). The chemo-resistance was associated with less than 25% of tumor shrinkage and 10% of tumor cell necrotic rate. All tissue specimens were paraffin-embedded for immunohistochemistry staining, or stored in liquid nitrogen for western blotting and real-time PCR analysis.

### Cell culture

Human osteosarcoma cell line MG63 was obtained from American Type Culture Collection (ATCC, Rockville, MD, USA) and cultured in RPMI1640 containing 10% FBS, 100 U/ml penicillin and 100 μg/ml streptomycin (all from Invitrogen, Grand Island, NY, USA) in a humidified atmosphere of 5% CO_2_ at 37 °C. The cisplatin-resistant MG63 (MG63/CDDP) cells were developed from the parental MG63 cells using an intermittent stepwise selection protocol as previously described [22, 23].

### Immunohistochemistry

Immunohistochemistry was used to detect the expression of Stim1 in all tissue specimens. The paraffin-embedded specimens were cut into 8-μm-thick sections, and deparaffinized and rehydrated in a graded series of alcohols. After antigen retrieval, the sections were incubated with 3% hydrogen peroxide for 10 min to block endogenous peroxidase activity, and then were immunostained with Stim1 antibody (1:50) (Santa Cruz Biotechnology, Inc., Paso Robles, CA, USA) at 4  °C overnight. After rinsing with PBS, the sections were incubated with horseradish peroxidase-conjugated secondary antibody (Zhongshan Jinqiao Co., Beijing, China) for 1 h. 3,3-diaminobenzidine tetrahydrochloride (DAB, Zhongshan Jinqiao Co.) was used as the substrate for the final visualization. Counterstaining was carried out with hematoxylin. The negative controls were processed in a same protocol with PBS instead of primary antibody. The sections were observed under a light microscope (Olympus, Tokyo, Japan).

### RNA extraction and real-time PCR

To quantify mRNA expression of Stim1 in tissue specimens, total RNA was prepared using Trizol reagent (Invitrogen) according to the manufacturer’s instruction. The isolated RNA was reverse transcribed using SuperScript II RT kit (Invitrogen). Stim1 mRNA expression was determined by real-time PCR on an ABI Prism^®^ 7300 (Applied Biosystems, Foster City, CA, USA) with SYBR Green Real Time PCR kit (Invitrogen). β-Actin was used as an internal control. The following primers were used: Stim1 sense 5′-GAATTGACAAGCCCCTGTGT-3′ and antisense 5′-ATGACTTCCATGCCTTCCAC-3′; β-actin sense 5′-GGCGGCACCACCATGTACCCT-3′ and antisense 5′-AGGGGCCGGACTCGTCATACT-3′.

### Western blotting analysis

Tissue specimens or cells were lysed using Western & IP Cell lysis Kit (Beyotime, Jiangsu, China) supplemented with protease inhibitor cocktail (Merck, CA, USA). The protein content was determined by BCA Protein Assay Kit (Beyotime). 40 μg of protein was separated by 8% SDS-PAGE and moved to polyvinylidene difluoride (PVDF) membranes (Millipore, Bedford, MA, USA). Blots were blocked and probed with antibodies against Stim1 (1:500), GAPDH (1:1000) (Santa Cruz Biotechnology), GRP78, CHOP, ATF4 (1:1000, Cell Signaling Technology Inc., Beverly, MA, USA) overnight at 4  °C. After washing, the blots were incubated with horseradish peroxidase-conjugated secondary antibodies for 1 h at room temperature and then visualized by ECL detection kit (Beyotime).

### Intracellular calcium ([Ca^2+^]_*i*_) measurements

[Ca^2+^]_*i*_ measurement was performed in 25 mm coverslip with cells after treatment indicated in the figure legends. Cells were loaded with 2 μM of Fura 2-AM (Invitrogen) in 2 ml of modified Krebs solution (in mM: 119 NaCl, 2.5 KCl, 1 NaH_2_PO_4_, 1.3 MgCl_2_, 20 HEPES, 11 glucose, 0.5 EGTA, pH 7.4) and incubated for 30 min at 37 °C. Ca^2+^ stores were passively depleted with 1 μM of thapsigargin (Tg) followed by addition of 1.8 mM of Ca^2+^. [Ca^2+^]*i* was measured using a wide-field inverted IX81 Olympus^®^ microscope with 340- and 380-nm wavelength filters, and analyzed with Olympus Cell^R software. A field of about 20 cells was imaged in each experiment, and the averaged fluorescence was assessed by counting 10 random fields per group in 6 independent experiments.

### Small interfering RNA-mediated gene silencing of Stim1

Stim1 knockdown in osteosarcoma cells was performed by transfection of human Stim1 siRNA (Applied Biosystems). The siRNA sequence targeting Stim1 was 5′-GGCTCTGGATACAGTGCTC-3′ (Stim1 siRNA) or 5′-GAGCACUGUAUCCAGAGCCTT-3′ (Stim1 siRNA-1). The non-specific siRNA oligonucleotides (Applied Biosystems) were used as a negative control. The siRNAs were transfected with Hiperfect Transfection Reagent (Qiagen, Valencia, CA, USA) according to the manufacturer’s instructions.

### Transfection

Full-length cDNAs for human STIM1 (NM_003156) were purchased from OpenBioSystems (Huntsville, AL, USA), and then were subcloned into the expression vector pSMCV (OpenBioSystems). The plasmid was confirmed by sequencing. To overexpress Stim1 in osteosarcoma, cells were transfected transiently with Stim1 plasmid for 24 h using the Lipofectamine Plus reagent (Invitrogen) in Opti-MEM medium (Invitrogen) according to the supplier’s instructions.

### Cell viability assay

MG63 cells or MG63/CDDP cells were seeded in 96-well plates at a density of 5 × 10^3^ cells/well overnight. After corresponding treatment indicated in the figure legends, CCK-8 reagent (Dojindo Laboratories, Kumamoto, Japan) was added and incubated for another 2 h at 37 °C. The absorbance of each well was measured at 450 nm by a plate reader (Bio-Tek, Winooski, VT, USA).

### Flow cytometry assay

MG63 cells or MG63/CDDP cells were harvested and cell apoptosis was determined by Annexin V-FITC/PI double-staining assay (Keygen, Jiangsu China) according to the manufacturer’s instructions. Apoptotic cells were counted with FAC-Scan flow cytometer (BD Biosciences, San Jose, CA, USA) and data were analyzed by CFlow Plus software (BD Biosciences).

### Statistical analysis

The results were presented as mean ± SEM. One-way analysis of variance (ANOVA) or the unpaired two-tailed Student’s *t* test was performed to compare the differences among groups using SPSS 15.0 statistical software (SPSS Inc., Chicago, IL, USA). Survival curves were estimated using Kaplan–Meier method and compared by log rank test. *P* value less than 0.05 was considered to indicate a significant difference.

## Results

### Increased Stim1 expression in chemo-resistant osteosarcoma tissues

**Fig. 1:**
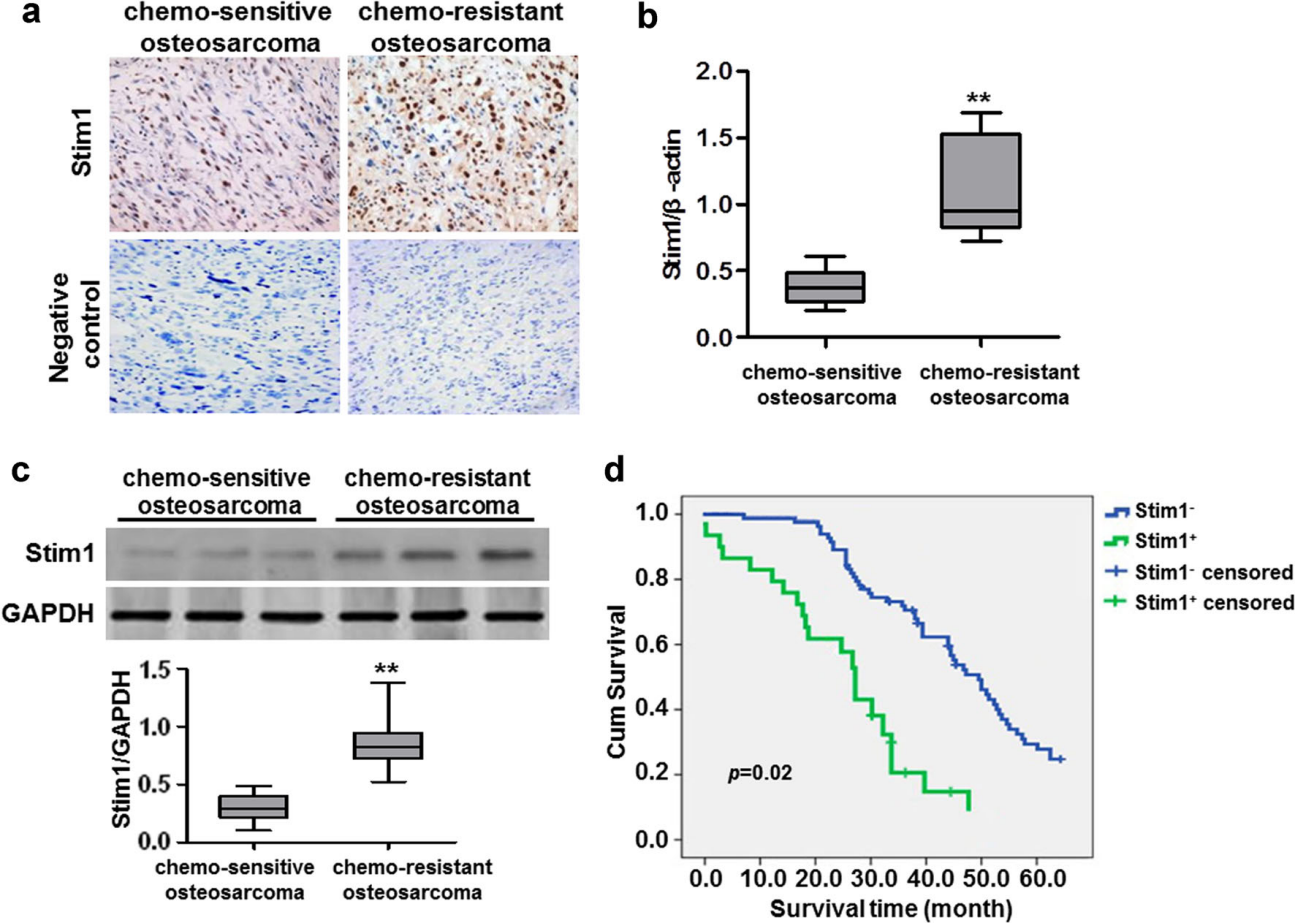
Stim1 is highly expressed in chemo-resistant osteosarcoma tissues. **a** Paraffin-embedded sections of chemo-sensitive osteosarcoma and chemo-resistant osteosarcoma tissues were collected. Expression of Stim1 was detected by immunohistochemistry. **b** Real-time PCR analysis to quantify the endogenous expression of Stim1 in chemo-sensitive tissue (*n* = 28) compared with chemo-resistant tissues (*n* = 32). **c** Stim1 protein expression was determined by Western blotting and quantitative results of Stim1 expression were presented. **P < 0.01 versus chemo-sensitive tissue. **d** Correlation of Stim1 expression and overall survival in patients with osteosarcoma

To determine the role of Stim1 in osteosarcoma, we first utilized immunohistochemistry to study the expression patterns of Stim1 in chemo-sensitive and resistant osteosarcoma tissues. The results showed that Stim1 was overexpressed in the chemo-resistant subjects who died within 3 years (Fig. 1a). Consistently, real-time PCR results revealed that the mRNA expression of Stim1 was also increased in chemo-resistant osteosarcoma tissues as compared with chemo-sensitive osteosarcoma tissues (Fig. 1b). Moreover, the protein expression of Stim1 determined by western blotting was similar to the immunohistochemistry and real-time PCR results (Fig. 1c), indicating that the increased Stim1 expression may be associated the chemo-resistance in osteosarcoma. Notably, Kaplan–Meier survival analysis revealed that Stim1-positive patients showed lower overall survival than those with Stim1-negative expression (*P* = 0.02 log-rank test) (Fig. 1d).

### Cisplatin decreases Stim1 expression and SOCE in cisplatin-sensitive osteosarcoma cells

**Fig. 2:**
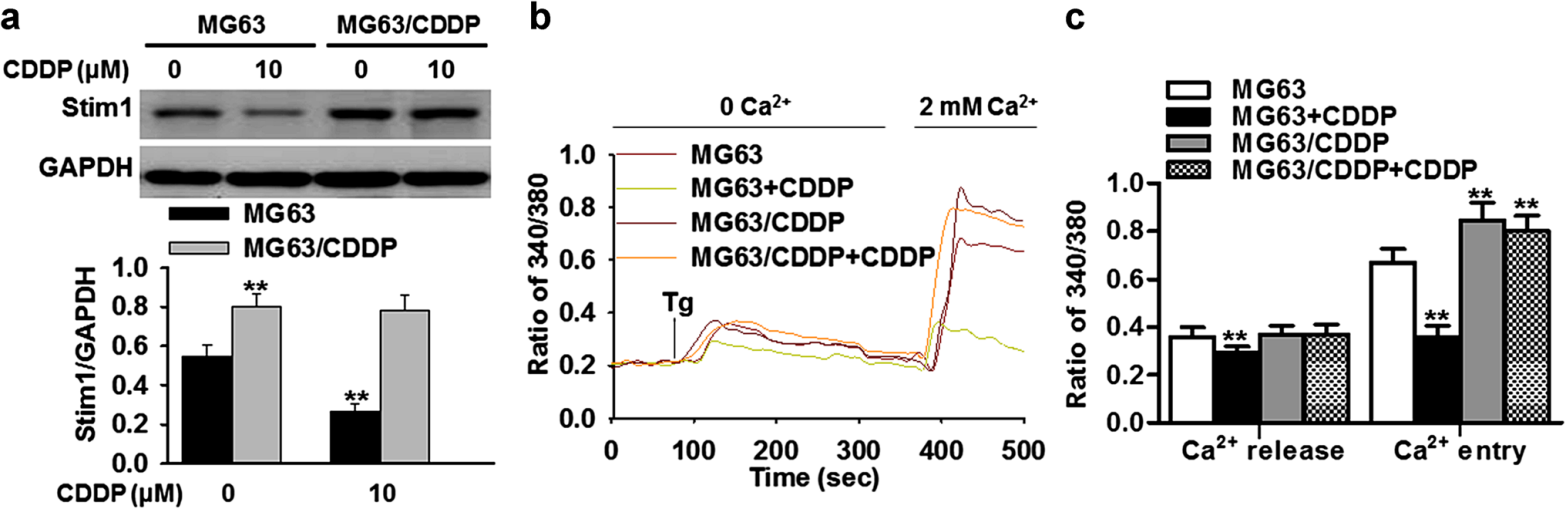
Cisplatin resistance is associated with higher expression of Stim1 in human osteosarcoma cells. **a** Cisplatin-sensitive (MG63) and resistant cells (MG63/CDDP) were treated with cisplatin (CDDP, 0 or 10 μM) for 24 h. Stim1 expression was examined by western blotting. **P < 0.01 versus MG63 cells, *n* = 6. **b** The cells were loaded with Fura 2-AM probe and subjected to Ca^2+^ imaging experiment. **c** Quantification of the thapsigargin (Tg)-induced SOCE amplitude in MG63 or MG63/CDDP cells treated with or without cisplatin (10 μM). **P < 0.01 versus MG63 cells, *n* = 5

To understand the link between Stim1 expression and cisplatin resistance in osteosarcoma cells, cisplatin-sensitive and resistant cells were treated with cisplatin for 24 h. In accord with the results from human tissue samples, higher expression of Stim1 was also found in cisplatin-resistant MG63/CDDP cells than in parent MG63 cells. Additionally, exposure of MG63 cells to cisplatin significantly decreased Stim1 expression, but had no effect in MG63/CDDP cells (Fig. 2a). As Stim1 is important for SOCE [12, 13], we next tested whether SOCE is altered upon cisplatin treatment. In the absence of extracellular Ca^2+^, cisplatin significantly inhibited thapsigargin (Tg)-induced Ca^2+^ release from ER store in MG63, but had no effect on MG63/CDDP cells. Notably, the SOCE, which was initiated by addition of external Ca^2+^ (2 mM), was dramatically increased in cisplatin-resistant MG63/CDDP cells compared with the parental cells. Furthermore, the Ca^2+^ influx was remarkably decreased by cisplatin treatment in MG63 cells, but not in cisplatin-resistant MG63/CDDP cells (Fig. 2b, c). Collectively, these results suggest that cisplatin inhibits Ca^2+^ release and entry at least partially via decreasing Stim1 expression.

### Cisplatin resistance is associated with inhibition of ER stress in osteosarcoma cells

**Fig. 3:**
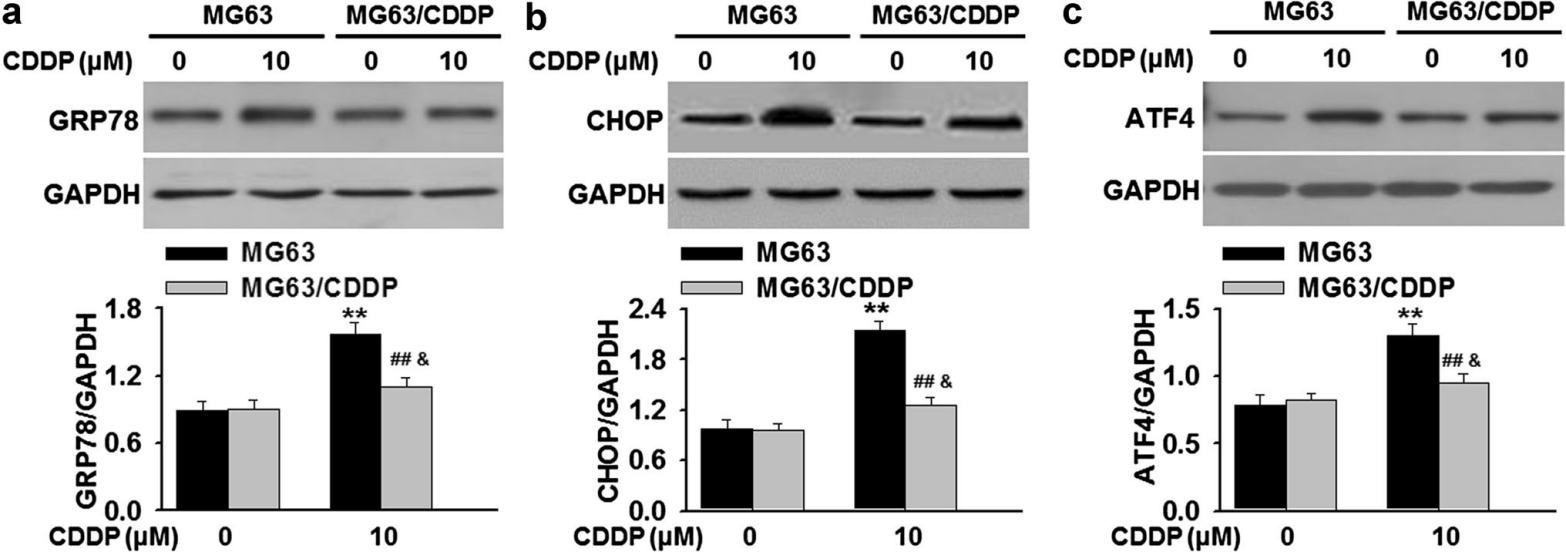
Cisplatin-induced ER stress in cisplatin-sensitive cells is more significant than in their resistant counterparts. **a**–**c** Cisplatin-sensitive (MG63) and resistant cells (MG63/CDDP) were treated with cisplatin (CDDP, 0 or 10 μM) for 24 h. The protein expressions of GRP78 (**a**), CHOP (**b**) and ATF4 (**c**) were detected by western blotting. **P < 0.01 versus MG63 cells; ^#*#*^ *P* < 0.01 versus MG63 cells + cisplatin; ^*&*^ *P* < 0.05 versus MG63/CDDP cells, *n* = 5

Since the shortage of ER Ca^2+^ may causes ER stress and apoptosis [21], we next evaluated if ER stress is involved in cisplatin resistance in osteosarcoma cells. MG63 and MG63/CDDP cells were treated with cisplatin for 24 h, respectively. Our results showed that cisplatin significantly increased the expression of molecular markers of ER stress, such as GRP78, CHOP and ATF4 in cisplatin-sensitive MG63 cells. Interestingly, the increase of GRP78, CHOP and ATF4 expression observed in MG63 cells was at least three times higher than in their counterparts, although cisplatin also resulted in a slight increase in these proteins in cisplatin-resistant MG63/CDDP cells (Fig. 3a–c). Taken together, these findings demonstrate that cisplatin-induced ER stress in chemo-sensitive osteosarcoma cells is more pronounced than in chemo-resistant cells.

### Stim1 knockdown sensitizes cisplatin-resistant osteosarcoma cells to cisplatin via enhancing ER stress-mediated apoptosis

**Fig. 4:**
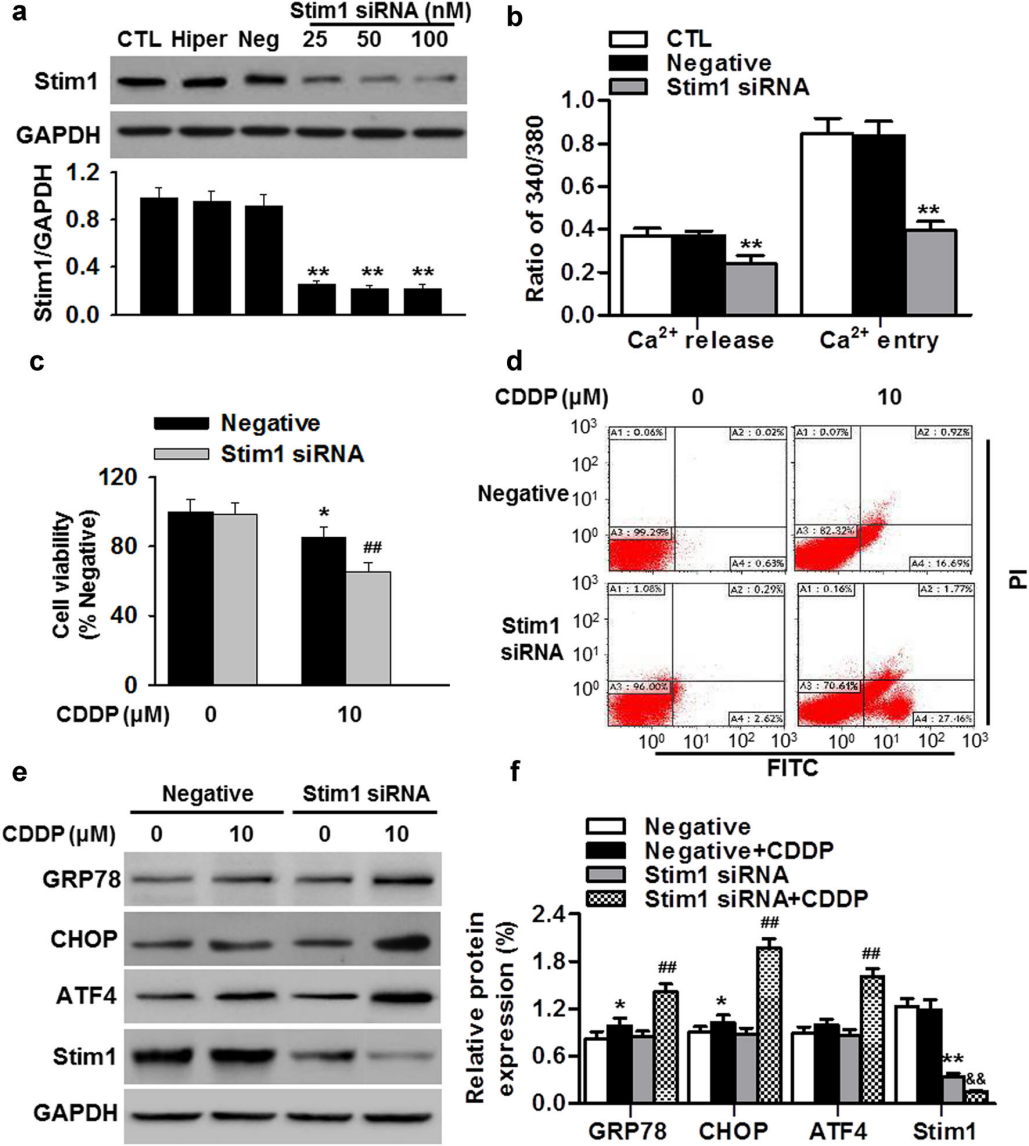
Knockdown of Stim1 increases chemo-sensitivity of cisplatin-resistant osteosarcoma cells to cisplatin treatment. **a** Cisplatin-resistant MG63/CDDP cells were transfected with negative siRNA (Neg), or different concentrations of Stim1-targeting siRNA (25, 50 and 100 nM) using Hiperfect reagent (Hiper) for 24 h. Stim1 expression was determined by western blotting. **P < 0.01 versus control, *n* = 4. **b** Quantification of the thapsigargin (Tg)-induced SOCE amplitude in MG63/CDDP cells transfected with negative or Stim1 siRNA. **P < 0.01 versus control, *n* = 6. **c** MG63/CDDP cells were transfected with negative or Stim1 siRNA (50 nM) for 24 h, followed by incubation of cisplatin (10 μM) for another 24 h. Cell viability was examined by CCK-8 assay. *P < 0.05 versus negative; ^#*#*^ *P* < 0.01 versus negative + cisplatin, *n* = 6. **d** Cell apoptosis was determined by Annexin V-FITC/PI staining followed by flow cytometry. **e** After treatments mentioned above, the protein expressions of GRP78, CHOP, ATF4 and Stim1 were examined by western blotting. **f** Densitometric analysis of the above protein expressions. *P < 0.05, **P < 0.01 versus negative; ^#*#*^ *P* < 0.01 versus negative + cisplatin; ^*&&*^ *P* < 0.01 versus Stim1 siRNA, *n* = 6

To elucidate whether Stim1 is involved in cisplatin resistance in osteosarcoma cells, cisplatin-resistant MG63/CDDP cells were transfected with Stim1 or negative siRNA for 24 h, followed by cisplatin incubation for 24 h. Down-regulation of Stim1 in MG63/CDDP was confirmed by western blotting (Fig. 4a). Expectedly, Tg-induced Ca^2+^ entry was significantly decreased by Stim1 down-regulation (Fig. 4b). Moreover, inhibition of Stim1 significantly enhanced cisplatin-induced the decrease in cell viability of MG63/CDDP cells (Fig. 4c). Similar results were observed when we used another siRNA sequence targeting Stim1, excluding the possibility that the effects of Stim1 down-regulation on cell viability were associated with the off-target effect (Figure S1). To test whether the decreased cell viability results from cell apoptosis, Annexin V-FITC/PI double staining flow cytometry was performed. The results showed that blockade of Stim1 markedly increased the percentage of apoptotic cells induced by cisplatin from 17.08 to 29.57% (Fig. 4d; Figure S2A). In addition, cisplatin-induced the increase of GRP78, CHOP and ATF4 expressions were more pronounced in Stim1 siRNA-treated cells than in negative siRNA-treated cells. Of note, although cisplatin treatment had no effect on Stim1 expression in negative siRNA-treated MG63/CDDP cells, it further decreased Stim1 expression in cells treated with Stim1 siRNA (Fig. 4e, f). Collectively, these results suggest that Stim1 knockdown promotes ER stress-mediated apoptosis in cisplatin-resistant osteosarcoma cells.

### Overexpression of Stim1 inhibits cisplatin-induced apoptosis in osteosarcoma cells

**Fig. 5:**
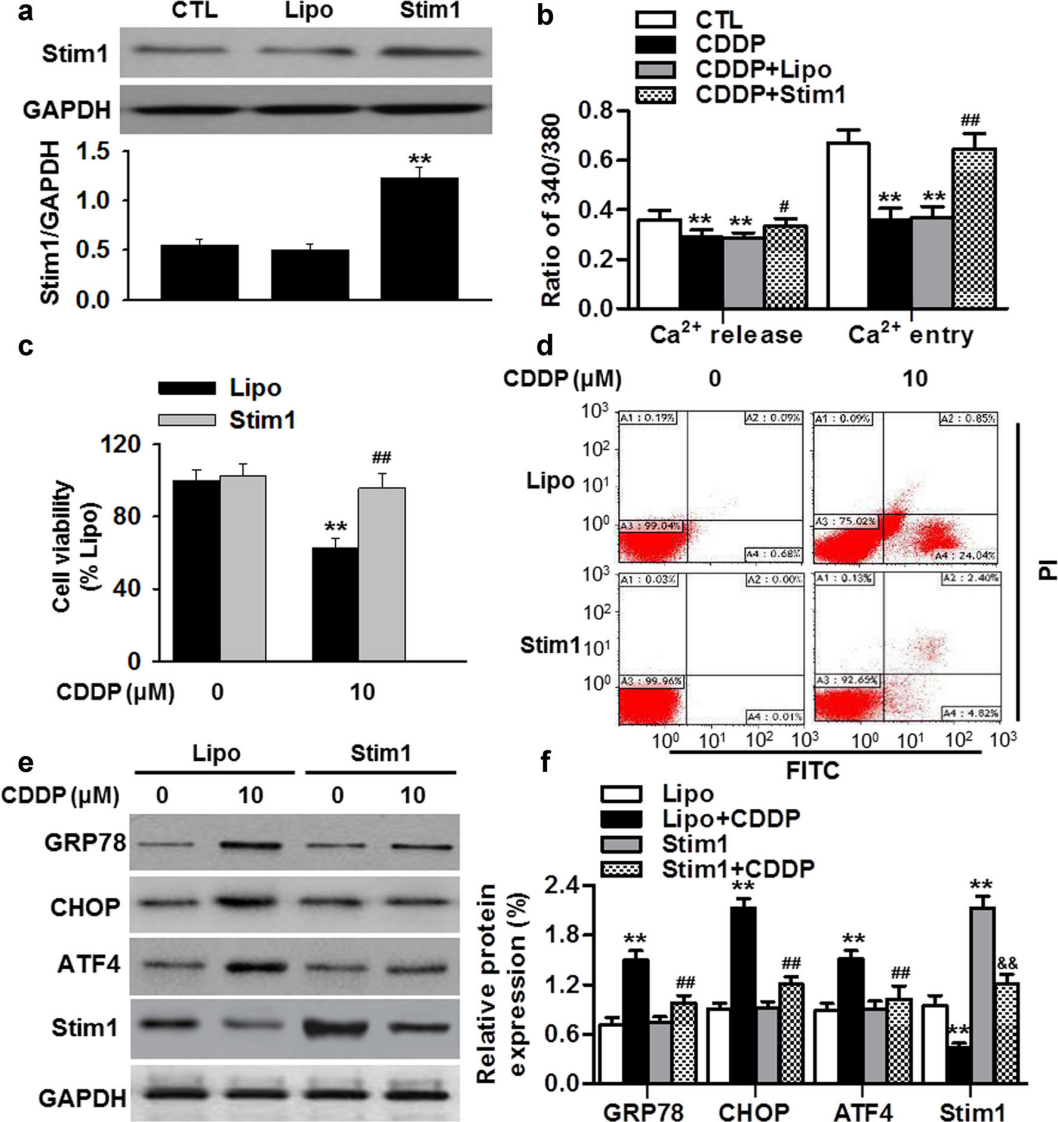
Overexpression of Stim1 decreases sensitivity of osteosarcoma cells to cisplatin treatment. **a** MG63 were transfected with Stim1 plasmid using Lipofectamine Plus reagent (Lipo) for 24 h. Stim1 expression was examined by western blotting. **P < 0.01 versus control, *n* = 4. **b** Quantification of the thapsigargin (Tg)-induced SOCE amplitude in MG63 cells transfected with Stim1 plasmid in the presence of cisplatin (10 μM). **P < 0.01 versus control; ^*#*^ *P* < 0.05, ^*##*^ *P* < 0.01 versus cisplatin alone, *n* = 6. **c** MG63 cells were transfected with Stim1 plasmid for 24 h and then were incubated with cisplatin for another 24 h. Cell viability was determined using CCK-8 assay. **P < 0.01 versus Lipo; ^*##*^ *P* < 0.01 versus Lipo + cisplatin, *n* = 6. **d** Cell apoptosis was analyzed by Annexin V-FITC/PI staining followed by flow cytometry. **e** After treatments mentioned in **c**, the protein expressions of GRP78, CHOP, ATF4 and Stim1 were determined by western blotting. **f** Densitometric analysis of the above protein expressions. **P < 0.01 versus Lipo; ^*##*^ *P* < 0.01 versus Lipo + cisplatin; ^*&&*^ *P* < 0.01 versus Stim1, *n* = 6

To further confirm whether cisplatin-inhibited Ca^2+^ influx (due to loss of Stim1 expression) is essential for ER stress-mediated apoptosis, we overexpressed Stim1 in cisplatin-sensitive MG63 cells. Up-regulation of Stim1 in MG63 cells was confirmed by western blotting (Fig. 5a). Ca^2+^ imaging showed that ectopic expression of Sitm1 restored cisplatin-induced the decrease of Ca^2+^ influx in MG63 cells (Fig. 5b). Furthermore, up-regulation of Stim1 significantly attenuated cisplatin-induced the decrease of MG63 cell viability (Fig. 5c). Similarly, the apoptosis of MG63 cells induced by cisplatin was also inhibited by Stim1 overexpression (Fig. 5d; Figure S2B). Finally, GRP78, CHOP and ATF4 expression that induce ER stress were all decreased in Stim1 overexpressing cells that were treated with cisplatin (Fig. 5e, f). Overall, the results demonstrate that elevated Stim1 expression restores cisplatin-inhibited Ca^2+^ influx and attenuates ER stress-mediated apoptosis in osteosarcoma cells.

## Discussion

In the present study, we observed up-regulation of Stim1 in chemo-resistant osteosarcoma tissues and cisplatin-resistant osteosarcoma cells. In addition, we demonstrated for the first time that cisplatin decreased Stim1 expression and SOCE in cisplatin-sensitive osteosarcoma cells, but not in cisplatin-resistant cells. Blockade of Stim1 and SOCE promoted ER-stress-mediated apoptosis induced by cisplatin, which conferred cisplatin resistance in human osteosarcoma cells. These findings provide important clues to the mechanisms involved in cisplatin resistance for osteosarcoma treatment.

Cisplatin is an efficient anti-cancer widely used for the treatment of various cancers [24]. However, frequent acquisition of cisplatin-resistant phenotypes is usually observed in cisplatin treatment, which has become a significant obstacle to develop better clinical use of cisplatin [6, 25]. It is urgent to understand the molecules involved in cell death and chemo-resistance resulting from cisplatin therapy. Here, to the best of our knowledge, our findings were the first to demonstrate that Stim1 expression could be up-regulated in chemo-resistant osteosarcoma tissues. In line with these results, we also found that Stim1 expression and SOCE were both increased in cisplatin-resistant MG63/CDDP cells compared to cisplatin-sensitive cells. These results, together with a previous study showing that Stim1 was overexpressed in liver cancer tissues compared with non-tumor tissues [15], indicate that Stim1 plays an important role not only in oncogenesis but also in chemo-resistance. Importantly, patients with Stim1-positive expression showed poorer survival than Stim1-negative patients, implying existence of Stim1 expression predicted worst survival and may as a critical prognostic indicator for osteosarcoma survival. Moreover, we found that cisplatin decreased Stim1 expression and SOCE in MG63 cells, but not in their resistant variants, further suggesting that increased Stim1 expression and subsequent SOCE may be a determinant of cisplatin resistance in osteosarcoma cells.

Intracellular Ca^2+^ serves as a vital role in regulating a plethora of cellular processes, including neural excitation, skeletal muscle contraction, endothelial cell proliferation and macrophage inflammation [7, 10, 11, 26]. Stim1 is the origin of SOCE, which has been implicated in several pathological processes of cancer, such as liver and breast cancer cell migration and metastasis [14, 15]. Previous studies suggested that Stim1 as well as SOCE contributed cancer cell apoptosis in response to different chemotherapeutic drugs. For example, Stim1 down-regulation was found to promote apoptosis induced by cisplatin in non-small cell lung cancer cells [8]. Recent study showed that pharmacological inhibition of SOCE sensitizes hepatocarcinoma cells to 5-fluorouracil treatment by increasing autophagic cell death [9]. Consistent with these results, we evidenced that knockdown of Stim1 sensitizes human osteosarcoma cells to cisplatin treatment, whereas overexpression of Stim1 increased chemo-resistance in cisplatin-resistant cells.

Since ER stress has been identified as a critical regulator of sensitivity to chemotherapy [27, 28], we next investigated the link between Stim1 and ER stress under cisplatin treatment. Here, our results showed that the increase of GRP78, CHOP, ATF4 expressions in cisplatin-sensitive MG63 cells was more pronounced than in the resistant cells in the presence of cisplatin, indicating the inhibition of ER stress may be required for cisplatin resistance. Moreover, knockdown of Stim1 further enhanced cisplatin-induced the increase of GRP78, CHOP and ATF4 in cisplatin-resistant MG63 cells, whereas overexpression of Stim1 was associated with decreased ER stress in cisplatin-sensitive cells. These results suggest that cisplatin decreases Stim1 expression and subsequent SOCE, a vital cell death mechanism for prevention of ER Ca^2+^ refilling, which ultimately results in ER stress-mediated apoptosis. It was worthy to note that induction of GRP78 may trigger pro-survival signaling that promotes resistance of chemotherapy, since it assists in the folding of nascent unfolded proteins [29, 30]. However, if the ER stress persists, the expression of CHOP and ATF4 that mediate the ER stress-induced apoptosis can be also induced, which may contribute to apoptosis overwhelming the pro-survival role of GRP78 [31]. Furthermore, although cisplatin resulted in a slight increase of molecular markers of ER stress in cisplatin-resistant MG63 cells, it did not have effect on Stim1 expression and SOCE, suggesting cisplatin-induced ER stress may be a multifactorial phenomenon. This should be further explored in the future.

In summary, we demonstrate that Stim1-mediated SOCE protects osteosarcoma cells from undergoing apoptosis in response to cisplatin. Knockdown of Stim1 effectively sensitizes cells to cisplatin via promoting ER stress-mediated apoptosis. Our study suggests that targeting Stim1 may be a therapeutic strategy for osteosarcoma chemotherapy.

## Electronic supplementary material

Below is the link to the electronic supplementary material.

Supplementary material 1 (DOC 87 kb)

## References

[1] Ward E, DeSantis C, Robbins A, Kohler B, Jemal A (2014). Childhood and adolescent cancer statistics, 2014. CA Cancer J Clin.

[2] Savage SA, Mirabello L, Wang Z, Gastier-Foster JM, Gorlick R, Khanna C (2013). Genome-wide association study identifies two susceptibility loci for osteosarcoma. Nat Genet.

[3] Sakamoto A, Iwamoto Y (2008). Current status and perspectives regarding the treatment of osteosarcoma: chemotherapy. Rev Recent Clin Trials.

[4] Bielack SS, Marina N, Ferrari S, Helman LJ, Smeland S, Whelan JS, et al. Osteosarcoma: the same old drugs or more? J Clin Oncol. 2008;26(18):3102–3 **(author reply 4–5)**. doi:10.1200/JCO.2008.17.1108. 10.1200/JCO.2008.17.1108 18565904

[5] Andrews PA, Howell SB (1990). Cellular pharmacology of cisplatin: perspectives on mechanisms of acquired resistance. Cancer Cells.

[6] Chu G (1994). Cellular responses to cisplatin. The roles of DNA-binding proteins and DNA repair. J Biol Chem.

[7] Abdullaev IF, Bisaillon JM, Potier M, Gonzalez JC, Motiani RK, Trebak M (2008). Stim1 and Orai1 mediate CRAC currents and store-operated calcium entry important for endothelial cell proliferation. Circ Res.

[8] Li W, Zhang M, Xu L, Lin D, Cai S, Zou F (2013). The apoptosis of non-small cell lung cancer induced by cisplatin through modulation of STIM1. Exp Toxicol Pathol.

[9] Tang BD, Xia X, Lv XF, Yu BX, Yuan JN, Mai XY (2016). Inhibition of Orai1-mediated Ca^2+^ entry enhances chemosensitivity of HepG2 hepatocarcinoma cells to 5-fluorouracil. J Cell Mol Med.

[10] Stiber J, Hawkins A, Zhang ZS, Wang S, Burch J, Graham V (2008). STIM1 signalling controls store-operated calcium entry required for development and contractile function in skeletal muscle. Nat Cell Biol.

[11] Stathopulos PB, Zheng L, Li GY, Plevin MJ, Ikura M (2008). Structural and mechanistic insights into STIM1-mediated initiation of store-operated calcium entry. Cell.

[12] McNally BA, Somasundaram A, Yamashita M, Prakriya M (2012). Gated regulation of CRAC channel ion selectivity by STIM1. Nature.

[13] Penna A, Demuro A, Yeromin AV, Zhang SL, Safrina O, Parker I (2008). The CRAC channel consists of a tetramer formed by Stim-induced dimerization of Orai dimers. Nature.

[14] Yang S, Zhang JJ, Huang XY (2009). Orai1 and STIM1 are critical for breast tumor cell migration and metastasis. Cancer Cell.

[15] Yang N, Tang Y, Wang F, Zhang H, Xu D, Shen Y (2013). Blockade of store-operated Ca(2+) entry inhibits hepatocarcinoma cell migration and invasion by regulating focal adhesion turnover. Cancer Lett.

[16] Schroder M, Kaufman RJ (2005). ER stress and the unfolded protein response. Mutat Res.

[17] Smedler E, Uhlen P (2014). Frequency decoding of calcium oscillations. Biochim Biophys Acta.

[18] Doutheil J, Treiman M, Oschlies U, Paschen W (1999). Recovery of neuronal protein synthesis after irreversible inhibition of the endoplasmic reticulum calcium pump. Cell Calcium.

[19] Shen L, Wen N, Xia M, Zhang YU, Liu W, Xu YE (2016). Calcium efflux from the endoplasmic reticulum regulates cisplatin-induced apoptosis in human cervical cancer HeLa cells. Oncol Lett.

[20] Zhang R, Wang R, Chen Q, Chang H (2015). Inhibition of autophagy using 3-methyladenine increases cisplatin-induced apoptosis by increasing endoplasmic reticulum stress in U251 human glioma cells. Mol Med Rep.

[21] Ma Y, Hendershot LM (2001). The unfolding tale of the unfolded protein response. Cell.

[22] Wu W, Li W, Zhou Y, Zhang C (2014). Inhibition of beclin1 affects the chemotherapeutic sensitivity of osteosarcoma. Int J Clin Exp Pathol.

[23] Yang C, Wu C, Xu D, Wang M, Xia Q. Astragaloside II inhibits autophagic flux and enhance chemosensitivity of cisplatin in human cancer cells. Biomed Pharmacother. 2016;81:166–75. doi:10.1016/j.biopha.2016.03.025. 10.1016/j.biopha.2016.03.025 27261591

[24] Kaufmann SH (1989). Induction of endonucleolytic DNA cleavage in human acute myelogenous leukemia cells by etoposide, camptothecin, and other cytotoxic anticancer drugs: a cautionary note. Cancer Res.

[25] Wu ZZ, Chao CC (2010). Knockdown of NAPA using short-hairpin RNA sensitizes cancer cells to cisplatin: implications to overcome chemoresistance. Biochem Pharmacol.

[26] Liang SJ, Zeng DY, Mai XY, Shang JY, Wu QQ, Yuan JN (2016). Inhibition of Orai1 store-operated calcium channel prevents foam cell formation and atherosclerosis. Arterioscler Thromb Vasc Biol.

[27] Ruwan Kumara MH, Piao MJ, Kang KA, Ryu YS, Park JE, Shilnikova K (2016). Non-thermal gas plasma-induced endoplasmic reticulum stress mediates apoptosis in human colon cancer cells. Oncol Rep.

[28] Ji GR, Yu NC, Xue X, Li ZG (2015). PERK-mediated autophagy in osteosarcoma cells resists ER stress-induced cell apoptosis. Int J Biol Sci.

[29] Jamora C, Dennert G, Lee AS (1996). Inhibition of tumor progression by suppression of stress protein GRP78/BiP induction in fibrosarcoma B/C10ME. Proc Natl Acad Sci USA.

[30] Dong D, Ni M, Li J, Xiong S, Ye W, Virrey JJ (2008). Critical role of the stress chaperone GRP78/BiP in tumor proliferation, survival, and tumor angiogenesis in transgene-induced mammary tumor development. Cancer Res.

[31] Schonthal AH (2013). Pharmacological targeting of endoplasmic reticulum stress signaling in cancer. Biochem Pharmacol.

