# Supplementary material for: Enhanced Stim1 expression is associated with acquired chemo-resistance of cisplatin in osteosarcoma cells
Source: Hum Cell. 2017 Mar 22;30(3):216–25. doi: 10.1007/s13577-017-0167-9 (PMC5486860; doi:10.1007/s13577-017-0167-9)
Supplement: Supplementary file 1 — Supplementary material 1 (DOC 87 kb) [file 13577_2017_167_MOESM1_ESM.doc]

**Enhanced Stim1 expression is associated with acquired chemo-resistance of cisplatin in osteosarcoma cells**

Xilong Sun^1^, Qiang Wei^2^, Jie Cheng^3^, Yanzhu Bian^3^, Congna Tian^2^, Yujing Hu^2^, Huijie Li^4^

^1^Department of Orthopaedics, ^2^Department of Nuclear Medicine, ^3^Department of Stomatology, Hebei General Hospital, ^4^Department of Orthopedics, The Third Hospital of Hebei Medical University, Shijiazhuang, Hebei, 050051, People’s Republic of China

**Running title:** Stim1 contributes cisplatin resistance

**Address correspondence to:** Huijie Li, Department of Orthopedics, The Third Hospital of Hebei Medical University, Shijiazhuang, No. 139, Ziqiang Road, Qiaoxi District, Hebei, 050051, People’s Republic of China. Tel: 86-0311-88602311, Fax: 86-0311-88602311. E-mail: lihj_hebmu@163.com

**Supplementary Figures**

Figure S1


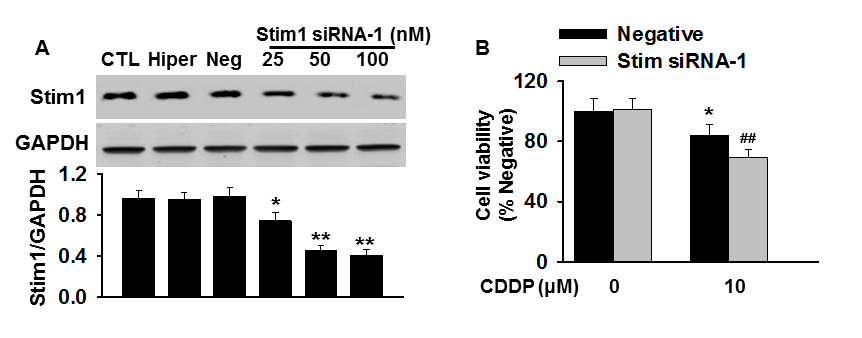


**Figure S1. Inhibition of Stim1 enhances cisplatin-induced the decrease in cell viability of MG63/CDDP cells.** (A) MG63/CDDP cells were transfected with negative siRNA (Neg), or different concentrations of another Stim1-targeting siRNA (Stim1 siRNA-1) (25, 50 and 100 nM) using Hiperfect reagent (Hiper) for 24 h. Stim1 expression was determined by western blotting. **P*<0.01, ***P*<0.01 versus control, n=4. (B) The cells were transfected with negative or Stim1 siRNA-1 (50 nM) for 24 h, followed by incubation of cisplatin (10 μM) for another 24 h. Cell viability was examined by CCK-8 assay. **P*<0.05 versus negative; *##P*<0.01 versus negative+cisplatin, n=6.

Figure S2


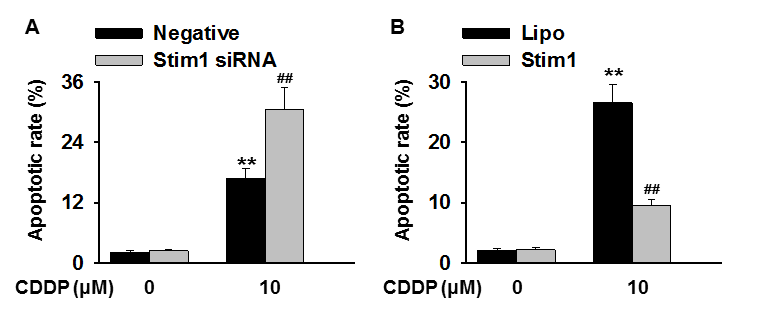


**Figure S2. Effects of Stim1 on cisplatin-induced apoptosis in MG63/CDDP cells or MG63 cells.** (A) MG63/CDDP cells were transfected with negative or Stim1 siRNA (50 nM) for 24 h, followed by incubation of cisplatin (10 μM) for another 24 h. Cell apoptosis was determined by Annexin V-FITC/PI staining followed by flow cytometry.Quantitative analysis of the percentage of apoptotic cells. ***P*<0.01 versus negative; *##P*<0.01 versus negative+cisplatin, n=6. (B) MG63 cells were transfected with Stim1 plasmid for 24 h and then were incubated with cisplatin for another 24 h. Cell apoptosis was analyzed by Annexin V-FITC/PI staining followed by flow cytometry.Quantitative analysis of the percentage of apoptotic cells. ***P*<0.01 versus Lipo; *##P*<0.01 versus Lipo+cisplatin, n=6.
